# Patient Preferences Regarding Chemotherapy in Metastatic Breast Cancer—A Conjoint Analysis for Common Taxanes

**DOI:** 10.3389/fonc.2018.00535

**Published:** 2018-11-21

**Authors:** Saskia Spaich, Johanna Kinder, Svetlana Hetjens, Stefan Fuxius, Axel Gerhardt, Marc Sütterlin

**Affiliations:** ^1^Department of Obstetrics and Gynaecology, University Medical Centre Mannheim, Heidelberg University, Mannheim, Germany; ^2^Department of Medical Statistics and Biomathematics, University Medical Centre Mannheim, Heidelberg University, Mannheim, Germany; ^3^Private Oncology Center Fuxius/Karcher, Heidelberg, Germany; ^4^Department of Gynaecology and Obstetrics, St. Hedwig-Klinik, Mannheim, Germany

**Keywords:** metastatic breast cancer, patient preference, shared decision-making, chemotherapy, conjoint analysis, taxane

## Abstract

**Background:** The purpose of this investigation was to explore patient perception regarding the importance of efficacy, toxicity, and logistics in the choice of regimen of taxane-based chemotherapy (CHT) for patients with metastatic breast cancer (MBC).

**Methods:** This dual-center study analyzed data of 100 women diagnosed with MBC, who were asked for their preferences regarding chemotherapy by means of conjoint analysis. Included attributes were progression free survival (PFS), application form, time and frequency, need of premedication, risk of alopecia, fatigue, febrile neutropenia, and neuropathy. Furthermore, participants completed a questionnaire about their personal and medical history. Regression analyses were performed to identify factors that influence patient preference in terms of specific treatment choice.

**Results:** Of 8 attributes, severe neutropenia was top priority for the majority of patients, followed by alopecia, neuropathy and PFS. When combining these patient preferences and the results of the questionnaire, patients' age as, well as, relationship status had significant impact on the importance of PFS. Moreover, longer travel time to the treatment center was significantly associated with preferences regarding PFS. Ranking by combination of respective part-worth values demonstrated nab-paclitaxel to be favored over paclitaxel and docetaxel.

**Conclusion:** Side effects of CHT and PFS prove to be critical factors for patients affecting choice of treatment in MBC with severe neutropenia being top priority, followed by alopecia, neuropathy, and PFS. Age, commute time, and relationship status were identified as significant determinants of patient preference. Total utility calculation by combination of part-worth values ranked nab-paclitaxel as the most preferable taxane.

## Introduction

Both curative and palliative treatment of breast cancer nowadays are characterized by a multimodal therapeutic approach and have led to significant improvements in survival (1, 2). At the time of initial diagnosis almost 5% of women with breast cancer already have metastatic disease (3) necessitating a palliative regimen with chemotherapy (CHT) as the central element of treatment. Taxane-based CHT may improve time to progression and survival (4, 5), but associated side effects constitute an important consideration when evaluating the best treatment concept for a patient (6). With regard to the latter, aspects of patient comfort and preference are gaining more attention in oncologic treatment of metastatic breast cancer (MBC). Perhaps more so than in other malignancies, the concept of shared decision-making is now well-established in the treatment of patients with breast cancer.

Risk of recurrence and overall survival have been elucidated as key parameters that guide patient preferences in regard to different therapeutic approaches (7). However, a critical goal of treatment in the palliative setting is to optimize quality of life (QoL), as therapy of MBC can seriously impair QoL (8, 9). The study of Lindley et al. demonstrates that patients who had severe disruptions in QoL are less willing to receive additional treatment for an extension of life compared with patients who experienced only little disruption to normal life (10).

The purpose of this investigation was to further explore and quantify patient preferences in terms of palliative chemotherapy by using the tool of conjoint analysis comparing common taxanes (Docetaxel, Paclitaxel, and nab-Paclitaxel) administered for MBC. Moreover, we attempted to identify different subgroups of patients, whose therapeutic choices—as evaluated in our conjoint analysis—are associated with sociodemographic factors, lifestyle and general mindset assessed in a separate questionnaire.

## Methods

Over 15 months a total of 111 patients with MBC were initially included in this dual-center study. These patients with an indication for a palliative treatment regimen were recruited at the University Medical Center Mannheim (UMM) and at the Oncology Center (OC), Outpatient Clinic Fuxius/Karcher in Heidelberg. All patients provided informed consent. The ethical approval for this study was obtained from the Ethics Committee II of Heidelberg University, Medical Faculty Mannheim (2014-536N-MA). Eleven patients had to be excluded because they did not understand the type of questioning and/or did not complete the conjoint analysis, so that full data sets were only available for 100 women.

### Questionnaire on socioeconomic/-demographic factors and general mindset

Participants obtained a questionnaire about their personal, professional, and medical history, as well as, their general mindset. In particular, women were asked to provide details about age, age at first diagnosis of BC, past medical history, distance to treatment facility, relationship status, level of education and working situation. In detail, questions covered, if their health had changed in the past year, if they had children and if they had to take care of somebody else. Finally, they were questioned about any prior personal experience with chemotherapies and possible side effects and if they had previous experience with glucocorticoids.

### Conjoint analysis

The tool of conjoint analysis was implemented to assess different attributes that drive individual patient preferences in regard to taxane-based chemotherapies. Generally, the technique of conjoint analysis is widely used in the medical and non-medical field for assessment of preferences and has been demonstrated to offer a valuable tool to elicit patient preferences or utilities for specific treatments (11–14). In regard to medical treatment, conjoint analysis has proven to be useful for preference elicitation mainly in cancer therapy (11, 13, 15, 16). By having participants evaluate alternatives and letting them choose between different combinations of attributes, the relative importance of each attribute can be deducted (17).

Attributes relevant to respective taxane-regimens assessed in our study were time to progression, application form and time, application frequency, need of premedication, alopecia, fatigue, febrile neutropenia, and polyneuropathy. While the attribute of alopecia does not have relevance for the differentiation between currently used taxanes because they do not actually differ in their high risk of alopecia (18–20), the attribute was nevertheless included in this study to allow for transfer of our results to potential future substances (such as cabazitaxel) that might differ in this respect.

In a second step, levels for each attribute were selected based on current literature (21–24) (Table 1). Attributes were described in non-medical terminology to optimize comprehension. Respective scenarios were created with Sawtooth software according to the manufacturers guidelines and current literature (25). As implementation of 8 attributes, each with 3 or 4 levels, gives rise to 300 possible combinations, each patient was confronted with a total of 20 randomly assigned conjoint sets.

**Table 1:** Characteristics of taxanes in treatment of metastatic breast cancer (MBC) used for conjoint analysis.

**Attributes**	Docetaxel^*^	Paclitaxel ^**^	Nab-Paclitaxel^***^	Cabazitaxel^****^
Progresion free survival	7.5 months (22)	9 months (23)	13 months (22)	–
Application time (i.v.)	60 min	180 min	30 min	60 min
Cycle	q21, d1	q7, d1	q28, d1,8,15	q21, d1
Premedication	+Dexamethasone	+Dexamethasone	None	No glucocorticoides
Alopecia	100% (18–20)	100% (18–20)	100% (18–20)	33% (24)
Neuropathy Grade 2-4	31% (22)	24% (sens.) (23) 9%(mot.) (23) 17% ^(21)^	40% (22)	–
Fatigue Grade 3-4	19% (22)	6% (23) 28% (21)	~3% (22)	–
Neutropenia Grade 3-4	94% (22)	10% (23) 42% (21)	44% (22)	67% (24)

Data refer to the following dosing regimens:

* *Docetaxel 100mg/m2 every 3 weeks*,

** *Paclitaxel 80mg/m2/weekly*,

*** *Nab-Paclitaxel 150mg/m2/weekly or 300mg/m2/weekly*,

**** *Cabazitaxel 20 mg/m2 + Capecitabine 825 mg/m2*.

In practice, after completing the questionnaire each patient sat with the investigator to be educated about the technique of conjoint analysis in general and the survey conduction at the notebook in particular. In this regard, they were provided with a couple of examples compatible to the ones used in the survey. At this point, all attributes and levels were explained in non-medical terminology. Specifically, each attribute itself was explained in detail, and (if applicable) grading, duration and severity of side effects were elaborated on. For the attribute of neuropathy, grade II-IV were presented as relevant side effects for patient's therapy. Patients were informed that grade III and IV would have impact on their activities of daily living and were likely to affect them for a longer period of time. However, moderate potential for rehabilitation was mentioned. Regarding the attribute of neutropenia, patients received information that chemotherapeutic agents generally confer leukopenia but that a relevant medical side effect would only arise from concurrent infection. Risk of infection was explained to be associated with severity of leukopenia. Patients were educated that only in moderate to severe infection in-hospital treatment with intravenous antibiotics and adjuvants would be required. Alopecia was described as hair loss that generally occurs shortly after the first course of CHT, while it usually resolves after completion of the last cycle of CHT.

Patients had to choose between 2 treatment options for each conjoint question (please refer to Supplemental Figure 1 for respective examples). Importantly, to reduce potential bias the conjoint survey did not include any drug names at all, but was only labeled with neutral terms (Option 1 and Option 2) from which patients chose their preferred alternative. Moreover, the investigator remained at the patient's side to answer any questions that would arise during conduction of the survey or help in case of technical difficulties. Sawtooth software was used again to analyse acquired data, yielding respective part-worth utilities and relative importance scores (RIS) for each attribute. These RIS for each attribute were then compared allowing for individual ranking of various attributes.

Finally, on the basis of the examined 8 attributes total utility of currently used taxanes was calculated by adding the respective part-worth utilities of each attribute, which most closely resembled those values published in recent literature for each respective taxane. This ranking allows for matching preferences of our study cohort with taxanes currently available for therapy of MBC.

### Univariate analyses of determinants

Integrating both the conjoint analysis and the results from the personal questionnaire, we sought to identify socioeconomic and—demographic factors, as well as, general attitudes in our cohort that had influenced patient preference toward any of the chemotherapeutics agents. Specifically, mean RIS values for each attribute and subgroup of patients were compared.

Statistical assessment was performed using SPSS (Version 22; SPSS Inc., USA) in cooperation with the Department of Statistics and Bioinformatics of the Medical Faculty Mannheim, Heidelberg University. Data are presented as mean ± standard deviation, respectively. The RIS values for each attribute and subgroup of patients were analyzed using univariate significance testing (*t*-test and ANOVA where applicable). A *p*-value below 0.05 was considered statistically significant.

## Results

**Figure 1:**
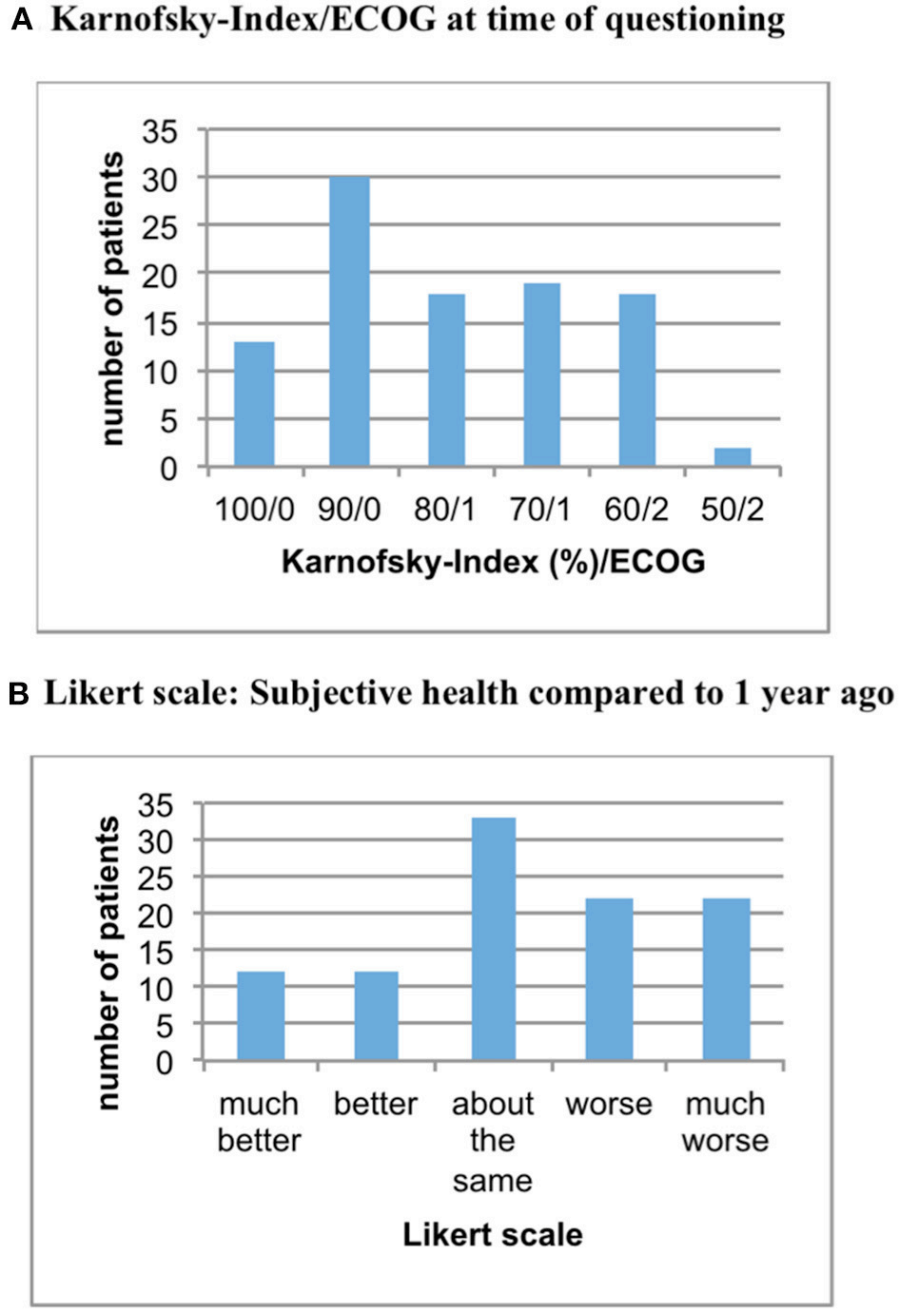
Functional status of patients at time of **(A)** and one year prior to **(B)** study conduction.

This dual-center study analyzed data of 100 patients. The age of women included ranged from 32 to 87 years (mean = 64 years). At time of initial diagnosis of breast cancer mean age was 55 years, with 29% of women having metastatic disease at this point. Mean duration of illness in our study cohort was 10.5 years. In terms of QoL, approximately 80% of women showed a Karnofsky-index of 70% or more (ECOG < 2) with the remaining women classified as Karnofsky 50–60% (= ECOG 2). About 4 in 10 patients reported progression of symptoms during the past year (see Figure 1).

### Lifestyle, mindset and sociodemographic factors

Detailed patient characteristics and sociodemographic factors, as well as, treatment experience are presented in Tables 2, 3.

**Table 2:** Demographic characteristics, overall (*n* = 100).

**Characteristics**
Age, mean + SD	64.4 ± 10.6 years
Age at time of initial diagnosis, mean + SD	55 ± 10.7 years
**TRAVEL TIME**	
≤ 25 minutes	59%
>25 minutes	41%
**EDUCATION**	
Low	37%
Mid	27%
High	36%
**WORKING STATUS**	
Currently employed	13
Unemployed	7%
Retired	80%
**MARITAL STATUS**	
Single	16%
Stable relationship	70%
Widowed	14%
**CHILDREN**	
Non	20%
1	24%
2	37%
3 or more	19%
**STATUS AS PRIMARY CAREGIVER (FOR)**	
No one	74%
Children	9%
Partner or parents	14%
Other	3%
**KARNOFSKY-INDEX**	
100%	13%
90%	30%
80%	18%
70%	19%
60%	18%
50%	2%
**SUBJECTIVE HEALTH COMPARED TO ONE YEAR AGO**	
Much better	12%
Better	12%
About the same	33%
Worse	22%
Much worse	21%

**Table 3:** Therapy experience and current therapy, *n* = 100.

**CHEMOTHERAPY**	
overall	**79%**
currently	**42%**
**CURRENT CHEMOTHERAPY**	
Taxanes	**18%**
Capecitabine	**12%**
Navelbine	**5%**
other	**8%**
Current endocrine therapy	51%
Current Her2/neu-Inhibitors	27%
Current Anti-VEGF-therapy	8%
**ADVERSE EVENTS RELATED TO CHEMOTHERAPY**	
Motor neuropathy	19% (*n* = 15)
Sensory neuropathy	49.4% (*n* = 39)
Fatigue	58.2% (*n* = 46)
Severe changes in blood count/parameters	19% (*n* = 15)
Alopecia	78.5% (*n* = 62)
Bone, muscle or joint pains	44.3% (*n* = 35)
None	8.9% (*n* = 7)
**TOLERANCE OF CHEMOTHERAPY**	
Good	45.6% (*n* = 36)
Moderate	31.6% (*n* = 25)
Poor	22.8% (*n* = 18)
Hospitalization required	16.5% (*n* = 13)
**TOLERANCE OF GLUCOCORTICOIDS**	
Good	65.5% (*n* = 42)
Moderate	23.4% (*n* = 15)
Poor	10.9% (*n* = 7)

*The bold values (percentages) denote the fraction of patients (with overall experience or current chemotherapy respectively), and which agent is currently applied if they are currently receiving chemotherapy*.

In terms of marital status, the majority (70%) of women were in a stable relationship, 14% of patients were widowed and the rest were single/divorced (16%). With regard to family, 80% of patients had children. However, only 26% of the women had to take care of somebody else in the family at the time of questioning.

The majority of patients had low or medium education (64%), while only 36% of women had a higher level of education.

Most of the women (80%) were retired at the time of questioning with only 13% of women still working. Pertaining to the dual-center character of our study, 51% of patients received medical treatment at the UMM, whereas 49% were patients at the OC Fuxius/Karcher. In terms of travel time, 59% of women stated that it took < 25 min commuting to the institution, while 41% of patients had to travel more than 25 min.

### Breast cancer experience

At the time of survey 42% of all patients were currently receiving CHT for MBC. Overall, 79% of the study population had already received CHT as part of their BC-treatment regimen in the past. The most common chemotherapeutics mentioned as part of the current chemotherapy were taxanes and capecitabine (Table 3). When asked about tolerance of previous treatment regimens, the majority (45.6%) had tolerated CHT well, whereas 22.8% of patients described poor tolerance. Hospitalization due to therapy occurred in 16.5% of cases. As part of their supportive therapy, 64% of women had taken oral glucocorticoids to reduce side effects, with 90% of these patients reporting moderate or good tolerance of this adjuvant.

### Conjoint analysis

**Figure 2:**
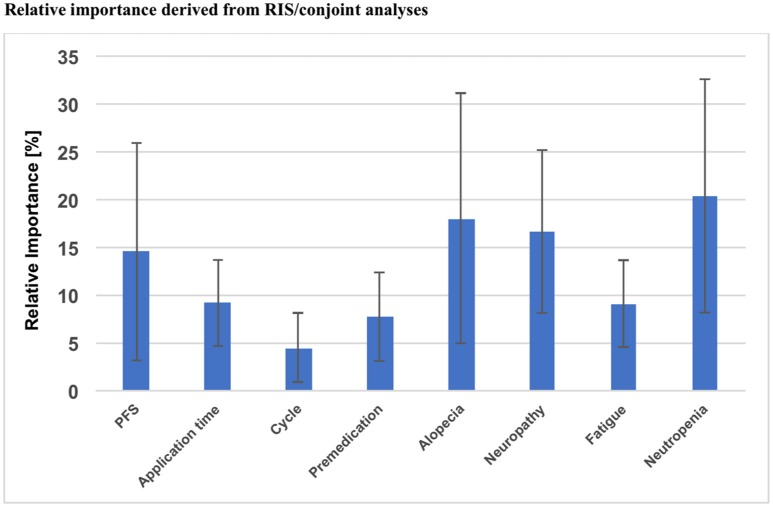
Overall results of the Conjoint Analysis: relative importance of each attribute, PFS, Progression free survival; RIS, Relative important score.

Using Sawtooth Software, preference weights (part-worth utilities) and relative importance scores (RIS) were calculated. On this basis, the attributes with the highest utility to patients were concerned with relevant side effects of therapy: avoidance of clinically significant neutropenia as top priority for the majority of patients (RIS = 20.35), followed by avoidance of alopecia (RIS = 18.02) and severe neuropathy (RIS = 16.79). Progression free survival was also crucial for patients, but only ranked fourth in this preference elicitation (RIS = 14.56). Severe fatigue (RIS = 9.10), application time (RIS = 9.14), necessity of premedication (RIS = 7.72) and number of application cycles (RIS = 4.41) represented considerably less relevant determinants of patient preference (Figure 2). A detailed list of RIS and respective part-worth values of the Conjoint Analysis are presented in Table 4.

**Table 4:** Relative Importance Score (RIS) and part-worth of the conjoint analysis.

	**Part-Worth**	**Range**	**RIS**
**Progression free survival**		105.48	**14.56**
7.5 months	−46.77	
9 months	−11.94	
13 months	58.71	
**Application mode and time**		41.06	**9.14**
Infusion 30 min.	16.10	
Infusion 60 min.	2.64	
Infusion 180 min.	−24.96	
Tablet at home	6.22	
**Cycle**		16.68	**4.41**
weekly	−8.34	
3-weekly	8.34	
**Premedication**		41.17	**7.72**
With steroids	−23.13	
Without steroids	5.10	
Not necessary	18.04	
**Alopecia**		121.86	**18.02**
100%	−61.35	
30%	0.85	
0%	60.51	
**Severe neuropathy**		118.8	**16.69**
30%	−47.00	
25%	−24.79	
5%	71.80	
**Severe fatigue**		39.97	**9.10**
30%	−12.99	
20%	−13.49	
5%	26.48	
**Severe neutropenia**		156.26	**20.35**
90%	−84.02	
60%	11.78	
30%	72.24	

*The bold values (percentages) denote the fraction of patients (with overall experience or current chemotherapy respectively), and which agent is currently applied if they are currently receiving chemotherapy*.

### Association of individual patient data and treatment preference

Another aim of this study was to determine whether these treatment preferences and RIS values were associated with any of the items assessed in the questionnaire on social and personal aspects of the patients' lives.

**Figure 3:**
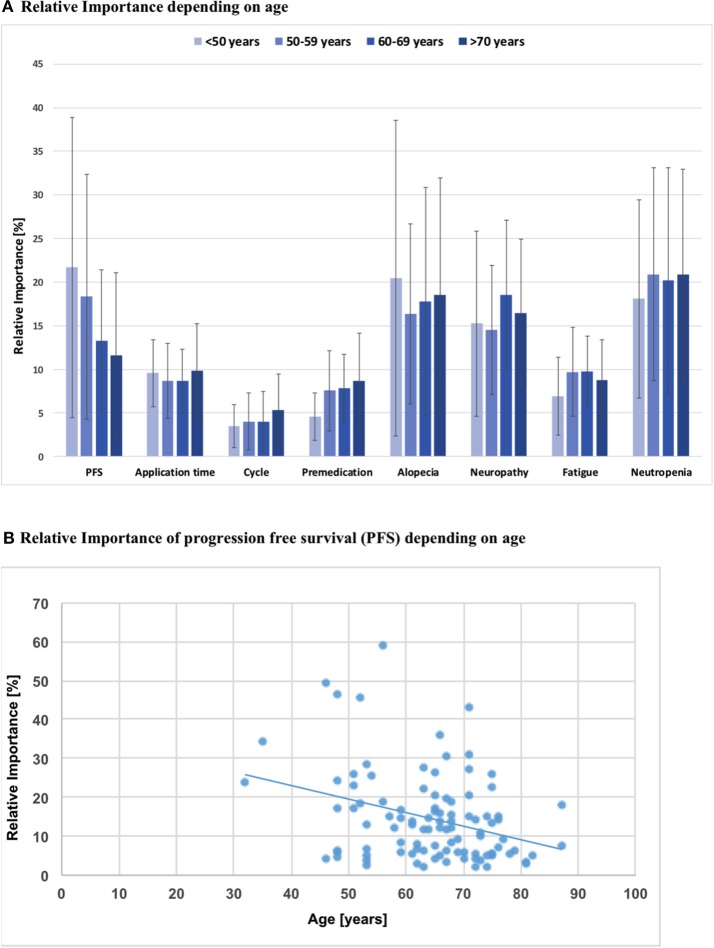
**(A)** Relative importance of each attribute depending on patients'age; PFS, Progression free survival; Intra-attribute RIS comparison does not show significant differences when stratified by age groups (*p* > 0.05, all n.s.). **(B)** Simple analysis of regression: Relative importance of PFS depending on patients' age; b = −0.35; *p* = 0.001.

Using univariate regression, a significant association was observed for age and importance of PFS in that the RIS of PFS was lower when the patient was older (ß = −0.348, *p* = 0.001). On the other hand, age did not have a significant influence on decisions regarding severe side effects of chemotherapy, when tested in a univariate regression model (Figure 3).

**Figure 4:**
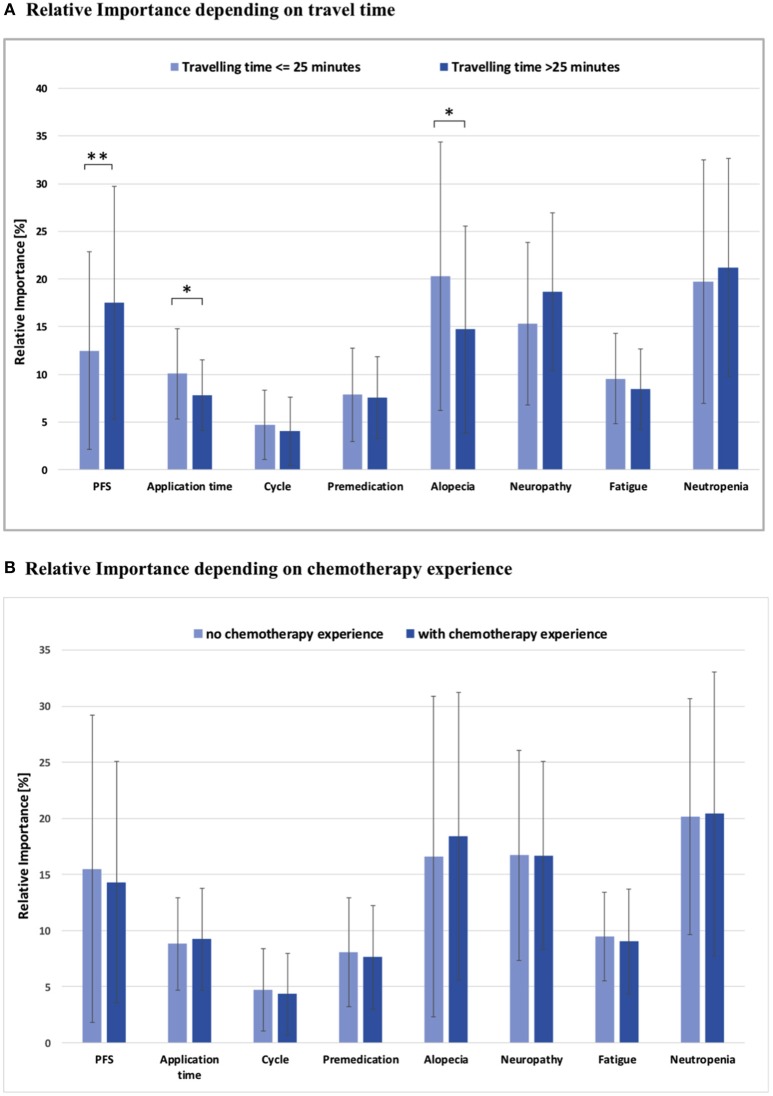
**(A)** Relative importance of each attribute depending on travel time to the medical center, ^*^p < 0.05, ^**^p < 0.01; all other comparisons not significant (*p* > 0.05). **(B)** Relative importance of each attribute depending on chemotherapy experience; PFS, Progression free survival; all comparisons not significant (*p* > 0.05).

Moreover, a significant association between travel time to treatment facility and PFS, application time and alopecia was observed (Figure 4A): Women with a commute time of more than 25 min showed a higher RIS for PFS than patients with less travel time (RIS 17.5 vs. 12.5; *p* < 0.01). For these patients with a longer distance to their respective treatment facility, time for application was not so important as for the other subgroup (RIS 7.8 vs. 10.1; *p* < 0.05). In addition, women with a longer travel time rather accepted alopecia than patients with a shorter distance to the treatment facility (RIS 14.7 vs. 20.3; *p* < 0.05).

Analysis of relationship status yielded a significant difference in terms of PFS, in that women who lived with a partner had a significantly higher RIS for PFS (RIS 16.1 vs. 10.9; *p* < 0.05).

Patient preference was remarkably consistent and did not vary significantly when stratified by previous treatment experience, as no substantial differences in the RIS of toxicities and CHT aspects were observed (Figure 4B).

In subgroup analyses no collective showed a higher RIS for alopecia than the group of patients who did not have prior experience with alopecia (RIS = 22.7). Comparing the RIS of patients with or without CHT in the past, there were no significant differences.

Regarding mode of application of CHT, women demonstrated preference for a short infusion at the treatment facility as compared to longer infusion times or regular domestic oral medication (Figure 4A). Moreover, they preferred longer cycles without premedication.

### Comparison of different taxanes

As the synopsis of this study, the data collected on patient preferences was used to rank the three most widely used taxanes (Docetaxel, Paclitaxel and nab-Paclitaxel). This calculation of total utility for each taxane on the basis of the investigated 8 attributes demonstrated nab-Paclitaxel as top preference with the highest total utility (14.4), followed by Paclitaxel (−95.3). Docetaxel had a (much) lower preference with a total utility of −264.8.

## Discussion

Breast cancer treatment has seen an impressive evolution in the past decades as therapeutic regimens have been augmented by addition and combination of new drugs, while at the same time the concepts of patient preference, shared decision-making and individualized therapy have steadily gained attention. From an economic perspective patient-centered approaches might also confer significant benefits, as estimations attribute about half of healthcare costs to decisions primarily driven by doctors and hospital supply rather than patient need and demand (26, 27). Therefore, focusing on patient preferences and elucidating reasons for individual choices in regard to certain therapies may strengthen economical but still effective approaches. Conjoint analysis and other questionnaires have been successfully used to determine these patient preferences in (breast) cancer therapy (6, 9). Importantly, implementation of conjoint analysis offers means to individualize a therapeutic strategy in order to carefully weigh risks and benefits on an individual level rather than extrapolate with population level data from clinical trials. Using conjoint analysis techniques may help in identification of trends in general patient preferences over time and detection of relevant shifts in culture. Interestingly, among other recent studies an excellent study by Ballinger et al. found patients to tend to favor toxicity concerns over treatment benefit (11, 13), while older studies often found survival benefit to be of supreme importance (16, 28–30). Clinical trials on population level generally focus on survival benefit as the primary endpoint and most critical aspect of therapy.

The results of our study show that patient preference in terms of favoring a chemotherapeutic regimen was mainly driven by avoidance of adverse effects: neutropenia as top priority (RIS 20.4), followed by alopecia (RIS 18.0) and neuropathy (RIS 16.8). Progression free survival—also considered crucial by patients—only ranked fourth in this preference elicitation (RIS 14.6). This is in congruence with data from previous studies (28–30), which could also show that the most influential factors in driving patient preferences for treatment were improved survival, risk of neutropenia and other side effects (28–30). Another important study by Smith et al. was published in 2014 and demonstrated treatment benefits to outweigh toxicity aspects (16). In contrast to our study, these above mentioned studies demonstrated that even a small incremental survival advantage had the highest relative importance seconded by side effects. This difference could be explained by the fact that the mean age of our study cohort was about 10 years higher as compared to the other studies. Furthermore, these younger patients often had a role as primary caregiver for children younger than 18 years of age (>35% in Smith's cohort vs. 9% in our cohort). This could possibly serve as an explanation, why (survival) benefits from treatment were shown to be critical in other studies (16). Accordingly, we could show that age was a significant determinant of treatment preferences in that the RIS of PFS was lower when patients were older (ß = −0.348, *p* = 0.001). Therefore, a decrease in age by 10 years led to a rise of the RIS of PFS by 3.5%. In addition, our study collective of MBC patients only will at least in part account for observed differences, as our study cohort of palliative patients differs from Beusterien's and Kuchuk's studies which included patients with all tumor stages (28, 30). As expected, women with an adjuvant therapy showed a higher RIS for PFS and accepted more side effects in these trials (28, 30).

A significant association between commute time to treatment facility and PFS, application time, as well as, alopecia was observed (Figure 4A): Women with a commute time of more than 25 min showed a higher RIS for PFS, while duration of each CHT session was not so important but significant. Furthermore, these patients rather accepted alopecia. It is tempting to speculate that these patients with greater distance to the treatment facility accepted more side effects and attached less importance to the application time because they consciously decided to go to a specialized center to optimize their treatment with focus on longer overall survival (31).

Patients living in a stable relationship also had a higher RIS for PFS. This might implicate that these patients weigh the risks and benefits differently, in that they appear to accept more side effects as the price for being able to spend as much time as possible with their partners. Other groups also found, that they would accept more side effects for longer overall survival (32–34).

Patient preference was remarkably consistent independent of prior experiences and did not vary significantly when stratified by previous treatment experience, as no substantial differences in the RIS of toxicities and CHT aspects were observed (Figure 4B). This is in congruence with data from previous studies, which could also show that determinants driving patient preference for treatment were generally independent of previous experience with CHT (28, 29). The only aspect that showed a trend toward differential preferences and ranking of potential side effects pertains to alopecia, which represented the overall second top priority for the majority of patients (RIS 18.0) in our study. The comparison of RIS values of patients with or without CHT in the past demonstrated that patients with prior CHT-based hair loss exhibit a lower RIS for this particular side effect. No subgroup showed a higher RIS for alopecia than the one without any prior experience of hair loss (RIS 22.7). A review of the literature shows markedly heterogenous reports concerning patient preference and alopecia. A study of DiBonaventura (29) demonstrated a distinct preference for treatment effectiveness and avoidance of alopecia. In contrast, investigations of Beusterien and Kuchuk (28, 30) suggested that alopecia does not have a significant impact. Another study by Al Batran and colleagues (35) also concluded that alopecia had no significant influence on QoL, but it deserves mentioning that most patients in this trial (73%) were men.

In unison, authors agree that the importance patients attribute to alopecia remains a very personal and still mostly unpredictable factor that should be addressed distinctly and individually when planning chemotherapy regimens (36). Furthermore, side effects that medical personnel might consider less relevant seem to have high priority for patients (37) and the question remains to which degree medical personnel should guide or sway these choices based on their expertise and experience. This may especially be true for putting alopecia into perspective, as patients that had never experienced hair loss before attribute a higher RIS to alopecia as compared to patients with a history of alopecia.

The literature offers a heterogeneous picture in terms of perceived severity of side effects of CHT and thus attributed RIS/ranks. In our study “severe fatigue” was considered the least relevant potential side effect for choosing a specific treatment, while Beusterien and DiBonaventura (28, 29) show that fatigue was more important than neutropenia to their study population. Consistent with our study, in the trial of Lloyd and colleagues neutropenia was also top priority for the majority of patients when asked about CHT and QoL.

At first glance, these observed differences in ranking the importance of side effects of chemotherapy seem surprising. However, they probably only reflect differences in study conduction, survey technique and patient education, as Lalla could adeptly show for MBC therapy in the past. In essence, Lalla et al. (6) investigated avoidance of side effects by primarily employing the “willingness-to-pay” technique. In principle, they could show that they acquired different results depending on the type of survey (6).

Another reason for differences in RIS/ranking could be derived from the level-effect: This effect can be developed if various attributes do not have the same number of values. Generally, the level effect will cause an attribute to show a higher RIS if the values are divided into more levels (38).

Consistent with previous studies, logistic aspects of therapy such as application time (RIS 9.1), necessity of premedication (RIS 7.7) and number of application cycles (RIS 4.4) had only minor influence on patient preferences and are much less controversially discussed (16, 28, 29).

As the synopsis of this study, total utility of each of the three most relevant taxane chemotherapeutics currently used (Docetaxel, Paclitaxel and nab-Paclitaxel) was calculated by combining RIS values of the 8 attributes examined in this study. This ranking of therapeutics by combined RIS values ranked nab-Paclitaxel top of the list with the highest total utility (14.4), followed by Paclitaxel (−95.3) and Docetaxel (−264.8),

Total utility calculation by addition of part-worth values must be interpreted very carefully due to the known limitations from this approach: only 8 attributes were examined in our study and these do not comprehensively characterize each chemotherapeutic agent. Furthermore, absolute differences in total utility do not reflect the dimension/degree by which one agent is considered superior over the other. Total utility calculation only allows for general ranking of substances. Moreover, this preference elicitation may only be used as guidance in a population that closely resembles the one from our study.

However, conjoint analyses and calculation of single or combined part-worth values offer a potential means to optimize the mosaic of the shared decision-making process by more closely monitoring and assessing patient preferences for given therapeutic interventions. Conjoint analysis also helps to weigh risks and benefits on an individualized level as compared to clinical trials that only yield population level data.

## Study limitations

Our study was not without limitations. First, the levels for the attributes were selected based on current literature and may not be reflective of individual experience. In addition, the participants of our study did not represent the full range of breast cancer patients (only MBC patients were included). Therefore, our preference elicitation must be interpreted with caution when applied to the general population of BC patients. Furthermore, our patient collective was rather old (mean age 64.4) as compared to other studies.

Moreover, patient information and explanation of attributes were not conducted according to a written standardized protocol. This might potentially cause a bias due to implicit connotations of the treating physician's explanations. Survey introduction, explanation of attributes and—maybe most importantly—the resulting discussion with the physician present at the time of survey may significantly impact a patient's preferences and the resulting effect cannot be dissected in this study. Future studies should therefore address this aspect carefully by optimisation of neutrality. These future studies should also systematically investigate this bias, as discussion of treatment options, risks and benefits with treating physicians itself is a cornerstone of shared decision-making.

## Conclusion

Our study on patient preferences regarding the most common taxanes (Docetaxel, Paclitaxel, and nab-Paclitaxel) validates that side effects of CHT and PFS are critical factors affecting choice of treatment in most MBC patients. For the majority, avoidance of neutropenia was the top priority, followed by alopecia, neuropathy, and PFS. However, treatment preferences significantly depended on age, as the RIS of PFS was highest among younger patients, causing it to be the top priority for choice of CHT agents in the group aged 50 or younger.

Apart from age as a determinant, our data show, that women in a stable relationship exhibited a higher RIS for PFS. Patients with a commute time of more than 25 min showed a higher RIS for PFS, while rendering application time and alopecia less important.

Total utility calculation by combination of part-worth values ranked nab-Paclitaxel as the most preferable CHT agent, followed by Paclitaxel and Docetaxel.

In essence, our data stress the notion that a critical goal of treatment in the palliative setting is to optimize quality of life (8, 9). Here, conjoint analysis may prove a very useful tool to individualize the therapeutic strategy by carefully weighing risks and benefits on an individual level rather than extrapolating with population level data from clinical trials.

## Author contributions

JK, SS, MS, and AG: Conception and design. MS and AG: Administrative support. JK, SS, AG, SF, and MS: Provision of study materials or patients. JK and SF: Collection and assembly of data. JK, SS, AG, SH, and MS: Data analysis and interpretation. SS, JK, and MS: Manuscript writing. SS, JK, SH, SF, AG, and MS: Final approval of manuscript.

### Conflict of interest statement

The authors declare that the research was conducted in the absence of any commercial or financial relationships that could be construed as a potential conflict of interest.

## Abbreviations

BC: breast cancer
BMI: body mass index
CHT: chemotherapy
MBC: metastatic breast cancer
RIS: relative importance score
PFS: progression free survival
QoL: quality of life.
