# Supplementary material for: Patient Preferences Regarding Chemotherapy in Metastatic Breast Cancer—A Conjoint Analysis for Common Taxanes
Source: Front Oncol. 2018 Nov 21;8:535. doi: 10.3389/fonc.2018.00535 (PMC6260130; doi:10.3389/fonc.2018.00535)
Supplement: Supplementary file 1 [file Table_1.DOCX]

Supplemental Figure 1.

Screenshot of a conjoint analysis survey question

| Please select the therapeutic option you prefer: | | |
| --- | --- | --- |
|  | Option 1 | Option 2 |
| Progression free survival | Statistically **progression** of tumour growth is stopped for **13 months**. | Statistically **progression** of tumour growth is stopped for **9 months**. |
| Application time | Infusion time of **30 minutes** | Infusion time of **180 minutes** |
| Cycle | You receive the medication **every week**. | You receive the medication **every 3 weeks**. |
| Premedication | Premedication is **necessary**, but **without cortisone/steroids**. | Premedication with **cortisone/steroids is necessary**. |
| Loss of hair | **No hair loss**. | **100%** chance of **hair loss** during chemotherapy. |
| Neuropathy | **30%** chance of **severe neuropathy**, with disturbance of sensitivity and movement disorders, like gait disorder, shaking, [tingling sensation](https://www.linguee.de/englisch-deutsch/uebersetzung/tingling+sensation.html) or numbness, resulting in [considerable impairment of activities of daily living (ADLs](https://www.linguee.de/englisch-deutsch/uebersetzung/considerable+restriction.html)). | **5%** chance of **severe neuropathy**, with disturbance of sensitivity and movement disorders, like gait disorder, shaking, [tingling sensation](https://www.linguee.de/englisch-deutsch/uebersetzung/tingling+sensation.html) or numbness resulting in [considerable impairment of activities of daily living (ADLs](https://www.linguee.de/englisch-deutsch/uebersetzung/considerable+restriction.html)). |
| Fatigue | **20%** chance of **extreme tiredness**, making it difficult to pursue activities of daily living. | **30%** chance of **extreme tiredness**, making it difficult to pursue activities of daily living. |
| Neutropenia | **90%** chance of a relevant decrease in the number of white blood cells, leading to an **increased susceptibility for infections**. | **30%** chance of a relevant decrease in the number of white blood cells, leading to an **increased susceptibility for infections**. |
|  | **○** | **○** |

Supplemental Figure 1. Screenshot of a conjoint analysis survey question.
